# Acute caffeine ingestion improves sport-specific and cognitive performance in elite badminton athletes

**DOI:** 10.3389/fnut.2025.1673882

**Published:** 2025-10-14

**Authors:** Liang Feng, Weiping Du, Xiaoting Wang, Hui Cao, Zhe Ji, Fang Li, Jianming Cao, Zhihui Zhou

**Affiliations:** ^1^School of Physical Education, Ningxia Normal University, Guyuan, China; ^2^Center for Sports and Health Research, Ningxia Normal University, Guyuan, Ningxia, China; ^3^School of Physical Education, Shaanxi Normal University, Xi'an, Shanxi, China; ^4^Department of Physical Education, North China Electric Power University, Beijing, China; ^5^School of Physical Education, Anhui Normal University, Wuhu, Anhui, China; ^6^School of Physical Education, He'nan Normal University, Xinxiang, He'nan, China; ^7^School of Competitive Sports, Beijing Sport University, Beijing, China

**Keywords:** caffeine, badminton performance, elite athletes, cognitive function, athletes

## Abstract

**Background:**

Caffeine, a widely used central nervous system stimulant, has been extensively studied for its potential to enhance exercise performance. However, systematic evaluations of its effects on badminton-specific performance remain limited, particularly in the domains of cognitive function and technical skills.

**Purpose:**

This study aimed to evaluate the acute effects of caffeine ingestion on cognitive, physical, and technical performance in elite badminton players.

**Methods:**

A randomized, double-blind, placebo-controlled crossover design was employed. Fifteen elite male badminton players holding a national first-class athlete certificate participated. Each ingested either caffeine (5 mg/kg body mass) or a placebo, followed by a 45-min absorption period and a 50-min standardized fatigue protocol. Participants then completed badminton-specific performance tests, including the Anticipation Skills Test (AST), Smash Accuracy Test (SAT), Shuttle Run Agility Test (SRAT), and Repeated Sprint Test (RST). A 7-day washout period was applied between conditions.

**Results:**

Significant treatment × time interaction effects were observed for anticipation accuracy (*F* = 4.992, *p* = 0.029), reaction time (*F* = 4.084, *p* = 0.048), and visual search frequency (*F* = 8.514, *p* = 0.005), indicating improved post-fatigue performance in the caffeine condition, whereas the placebo condition declined or remained unchanged. In terms of physical performance, the caffeine group demonstrated superior overall agility in the SRAT (*F* = 4.097, *p* = 0.048) and showed an anti-fatigue effect in the RST (*F* = 5.283, *p* = 0.025). However, caffeine ingestion did not significantly affect smash accuracy (*p* = 0.942) or ratings of perceived exertion (RPE; *p* = 0.917).

**Conclusion:**

Acute ingestion of 5 mg/kg caffeine significantly enhanced cognitive and physical performance under fatigue in elite badminton players, while exerting no apparent effect on fine technical skills.

## 1 Introduction

Caffeine, the primary psychoactive component of coffee and tea, has been extensively investigated for its effects on cognitive function and overall health. A large body of literature indicates that moderate caffeine intake can significantly enhance attention, reaction capacity, and overall mental state (1, 2). For example, Wu et al. (3) reported that caffeine ingestion effectively improved self-reported alertness as well as performance across multiple cognitive tasks, supporting its efficacy as a cognitive enhancer. Furthermore, Ricupero and Ritter. (4) summarized that caffeine is regarded as the principal ingredient responsible for enhancing attention, concentration, and reducing fatigue, consistent with the combined effects of other constituents.

At the health level, moderate caffeine intake has been shown to be associated with multiple benefits, including reduced all-cause mortality and improved metabolic function (5, 6). However, compared with these public health effects, the ergogenic role of caffeine in sports performance holds greater research and practical value, which is the primary focus of the present study.

Beyond its general health benefits in the wider population, the ergogenic effects of caffeine as a performance enhancer have been extensively studied and widely recognized. Moderate intake (3–6 mg/kg body mass) is generally applicable across most forms of exercise and has been shown to effectively enhance both anaerobic capacity and endurance (7–9). Moreover, research has demonstrated that caffeine ingestion significantly increases mean and peak power output, showing clear advantages compared with placebo (7, 10). In racket sports, caffeine has also been reported to facilitate sport-specific skills. For instance, in tennis skill tests, a 6 mg/kg dose significantly improved hitting success, while a lower 3 mg/kg dose enhanced shot accuracy (7, 9, 10). These effects may be attributed to mechanisms such as enhanced motor unit recruitment, increased intramuscular calcium availability, delayed perception of fatigue, and improved psychological state (11, 12). Furthermore, caffeine has been shown to improve athletes’ mood and motivation, thereby further supporting performance optimization under high-intensity conditions (10, 12).

Badminton is a fast paced and highly competitive racket sport that places substantial demands on both physical and cognitive performance (13). Matches involve numerous high-intensity actions such as sprints, jumps, and smashes, with players covering an average distance of up to 6.4 km nearly twice that reported in tennis. Furthermore, shuttlecock speeds can reach up to 70 m/s, requiring athletes to perform perceptual judgments and tactical responses within extremely short time frames (14). To maintain technical stability under such conditions, players must sustain high levels of concentration and rely on efficient visual search strategies and critical kinematic cues (e.g., shoulder, elbow, and racket position) to ensure shot accuracy (15, 16). Therefore, in the context of intense competition and rapid decision-making, investigating nutritional strategies such as caffeine ingestion to preserve physical output and cognitive efficiency holds important practical relevance.

Although several studies have examined the ergogenic effects of caffeine in racket sports, findings remain somewhat limited. For example, compared with carbohydrate supplementation or physical cooling interventions, caffeine has demonstrated more pronounced improvements in serve velocity (7, 9, 10). In badminton and related disciplines, caffeine ingestion has been shown to significantly enhance grip strength, running speed, and sprint frequency. Moreover, improvements in squat jump and countermovement jump performance have also been reported (17, 18). However, most of these investigations have primarily focused on the physiological aspects of badminton performance, while the potential benefits of caffeine on cognitive functions (e.g., reaction time, visual search, anticipation) and sport-specific technical skills remain insufficiently explored.

Therefore, to comprehensively evaluate the effects of caffeine on badminton-specific performance, we designed a randomized, double-blind, placebo-controlled crossover trial. The purpose of this study was to examine whether acute ingestion of caffeine (5 mg/kg body mass, 45 min before simulated match play) could enhance cognitive, physical, and technical performance in elite badminton players under fatigue. We hypothesized that caffeine would significantly improve cognitive abilities (e.g., anticipation accuracy, reaction time, and visual search efficiency) and physical performance (e.g., agility and repeated sprint ability), whereas its impact on technical skills (e.g., smash accuracy) would be relatively limited.

## 2 Materials and methods

### 2.1 Participants

A total of 15 healthy elite male badminton players, all holding the Chinese National First Class Athlete Certificate, were recruited for this study (age: 20 ± 2.01 years; height: 1.78 ± 0.06 m; body mass: 68.4 ± 7.1 kg; mean ± SD). All participants had extensive competitive training experience, with an average training history of 11.5 ± 3.2 years.

The sample size was determined with reference to previous crossover-design studies in sports nutrition, where the acute ergogenic effects of caffeine typically fall within the moderate-to-large range (Cohen’s d ≈ 0.5–0.7) (17, 19, 20). With a significance level of *α* = 0.05 and a paired design, an effect size of d = 0.6–0.7 requires approximately 12–20 participants to achieve a statistical power of 0.80–0.95. Accordingly, the inclusion of 15 athletes in the present study was sufficient to ensure adequate statistical power.

Given the elite competitive level of this population and the difficulty of recruitment, the sample size was relatively limited but ensured high homogeneity and professionalism of participants, thereby enhancing both interpretability and external validity of the findings. Notably, all athletes were classified as low habitual caffeine consumers, defined as <50 mg/day. Previous systematic reviews and meta-analyses have shown that, within conventional ergogenic doses (3–6 mg/kg), sex has limited influence on the acute effects of caffeine (21). Therefore, sex was not considered a confounding factor in this study.

Exclusion criteria included: (1) the presence of musculoskeletal pain or any other condition that could potentially affect exercise performance; and (2) the use of any pharmacological treatment within the past 3 months.

This study was approved by the local ethics committee (approval number: 2020084H). All participants provided written informed consent prior to participation, and all procedures were conducted in accordance with the Declaration of Helsinki.

### 2.2 Study design

**Figure 1:**
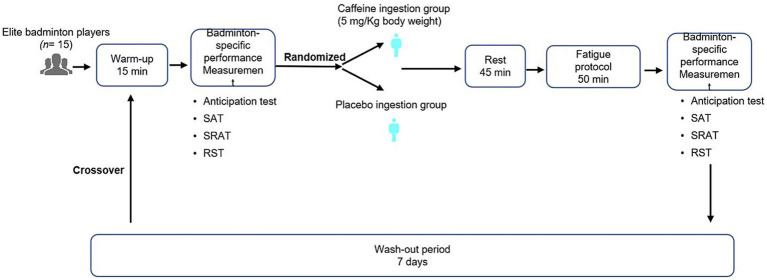
Experimental design flowchart.

This study employed a randomized, double-blind, placebo-controlled, crossover design. All participants first completed a 15-min standardized warm-up, followed by badminton-specific baseline testing (Figure 1). Participants were randomly assigned to one of two intervention sequences:

CAF → PLA: caffeine (CAF) ingestion in the first period, followed by placebo (PLA) ingestion in the second period. PLA → CAF: placebo ingestion in the first period, followed by caffeine ingestion in the second period.

Randomization sequences were generated using a computer-based random number table and implemented through sealed, opaque envelopes by an independent researcher not involved in the trial. Allocation concealment was maintained, and both participants, testers, and data analysts were blinded to group assignment.

The caffeine intervention was administered at a dose of 5 mg/kg body mass (Key Pharmaceuticals, Australia), based on the following rationale: First, previous systematic reviews and meta-analyses have demonstrated that 3–6 mg/kg is the most commonly used and well-supported effective range, capable of significantly improving strength, endurance, sprint performance, and cognitive outcomes (19). Second, both systematic reviews and the position stand of the International Society of Sports Nutrition (ISSN) indicate that higher doses (≥6–9 mg/kg) may yield additional benefits for certain performance measures, but are also associated with a substantially increased incidence and severity of side effects, making them unsuitable in competitive settings (21, 22). Therefore, a dose of 5 mg/kg was selected in the present study as it provides sufficient ergogenic effects while maintaining an acceptable safety profile.

The placebo tablets were identical in appearance, size, and taste to the caffeine tablets and were composed of soluble dietary fiber (Shengtang Pharmaceutical, Beijing, China). All participants ingested the assigned treatment 45 min before testing to ensure adequate absorption.

To minimize potential confounders, participants were instructed to abstain from high-intensity training for 48 h prior to testing, avoid any caffeine-containing foods or beverages for at least 24 h, and maintain their habitual dietary patterns. Regular sleep (7–9 h) was required the night before each trial, and both diet and sleep were monitored using self-report questionnaires to control for potential influences.

During each experimental period, participants completed a 50-min standardized badminton-specific fatigue protocol designed to replicate the high-intensity competitive demands of real matches. Immediately after the protocol, participants performed the same battery of badminton-specific performance tests as conducted at baseline, in a fixed sequence. Water was permitted ad libitum throughout the procedures.

To avoid potential carryover effects, a 7-day washout period was implemented between conditions, which is sufficient to ensure complete clearance of caffeine from the body. Both trials were conducted at the same facility and scheduled at the same time of day to maintain consistency in environmental conditions.

To further ensure the internal validity of the study, both blinding efficacy and compliance were assessed.

Blinding check: At the end of each experimental period, participants completed a questionnaire indicating whether they believed they had ingested caffeine or placebo. The responses were used to evaluate the effectiveness of the double-blind procedure.

Compliance check: On the day prior to each trial, participants filled out a dietary and lifestyle record to confirm abstinence from caffeine-containing foods and beverages. On the test day, supplement ingestion was directly supervised by the research staff, and compliance was continuously monitored throughout the protocol to ensure strict adherence to the study procedures.

In the blinding assessment, the overall correct guess rate across both periods was 53.3% (95% CI: 34.3–71.7%). An exact binomial test indicated no significant difference compared with the 50% chance level (*p* = 0.82), suggesting that participants were unable to distinguish between caffeine and placebo, and therefore the blinding was considered effective.

### 2.3 Experimental procedures

#### 2.3.1 Badminton-specific anticipation skills test (AST)

**Figure 2:**
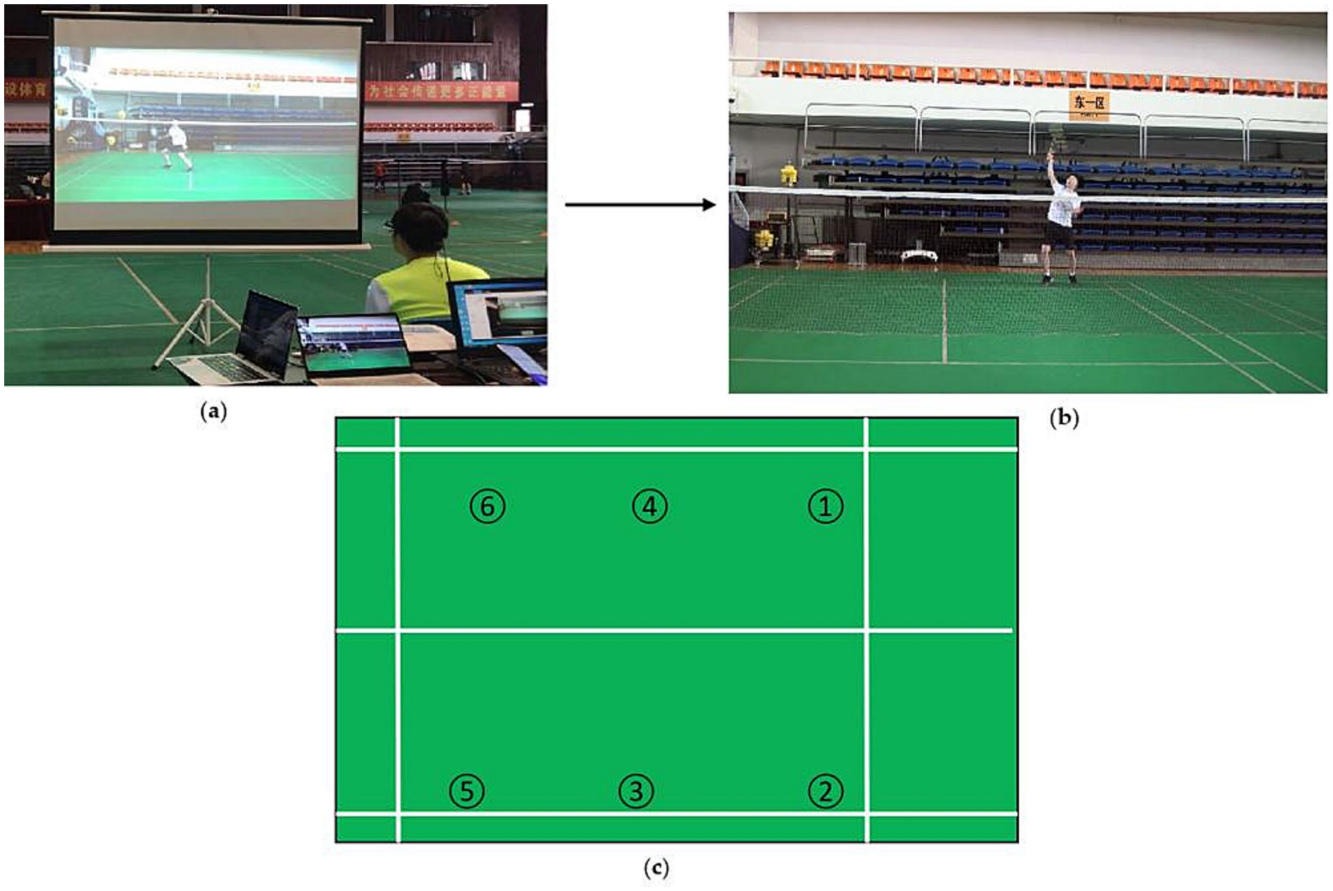
Schematic illustration of the badminton-specific anticipation test. **(a)** Experimental setup of the anticipation task. **(b)** Each video clip lasted 2,000 ms, showing a player moving from the mid-court to the backcourt to execute a stroke. The video was paused at the moment of racket–shuttle contact, after which participants were required to predict the shuttle’s landing area on the opponent’s court. **(c)** Response mapping for shuttle landing zones. Numbers 1–6 represent the right forecourt, left forecourt, right midcourt, left midcourt, right backcourt, and left backcourt, respectively.

This test was designed to evaluate the cognitive anticipation ability of badminton players (Figure 2). Participants were required to predict the trajectory of the shuttle based on video clips, which were recorded from a first-person perspective simulating matches played by national-level badminton athletes. Responses were made using a small keyboard connected to a computer, where keys 1, 2, 4, 5, 7, and 8 corresponded to six landing zones: left high clear, right high clear, left smash, right smash, left drop, and right drop, respectively. The system automatically recorded both reaction time and anticipation accuracy.

Specifically, the test consisted of three stroke types (clear, drop, and smash), with 12 video clips for each type. Each clip lasted 2,000 ms and was projected onto a screen (2.29 m × 3.05 m) positioned approximately 3 m in front of the participant, thereby providing a realistic match-viewing perspective.

During the test, participants wore a portable eye tracker (aSeeGlasses, aSeeStudio; 7Invensun, Beijing, China) to record eye-movement data at a sampling rate of 120 Hz. The eye-tracking data were processed and analyzed using the manufacturer’s analysis system. Areas of interest (AOIs) included the opponent’s head, arms, racket, and trunk as presented in the video. A fixation was defined as a gaze maintained for more than 100 ms (23).

Visual search rate was calculated as the number of fixations divided by the total time, with a higher ratio indicating lower search efficiency (15, 24). The final fixation duration was defined as the length of the last fixation on the screen before the video ended; shorter durations were interpreted as reflecting lower processing efficiency (25).

#### 2.3.2 Smash accuracy test (SAT)

This test consisted of 20 forehand smash attempts (10 straight-line and 10 cross-court), which are common offensive techniques in badminton. Players were instructed to perform each smash in the same manner as in official competition, using standardized shuttlecocks (Aerosensa 50, Yonex®, Tokyo, Japan). The target was a rectangular zone (250 cm in length × 80 cm in width).

**Figure 3:**
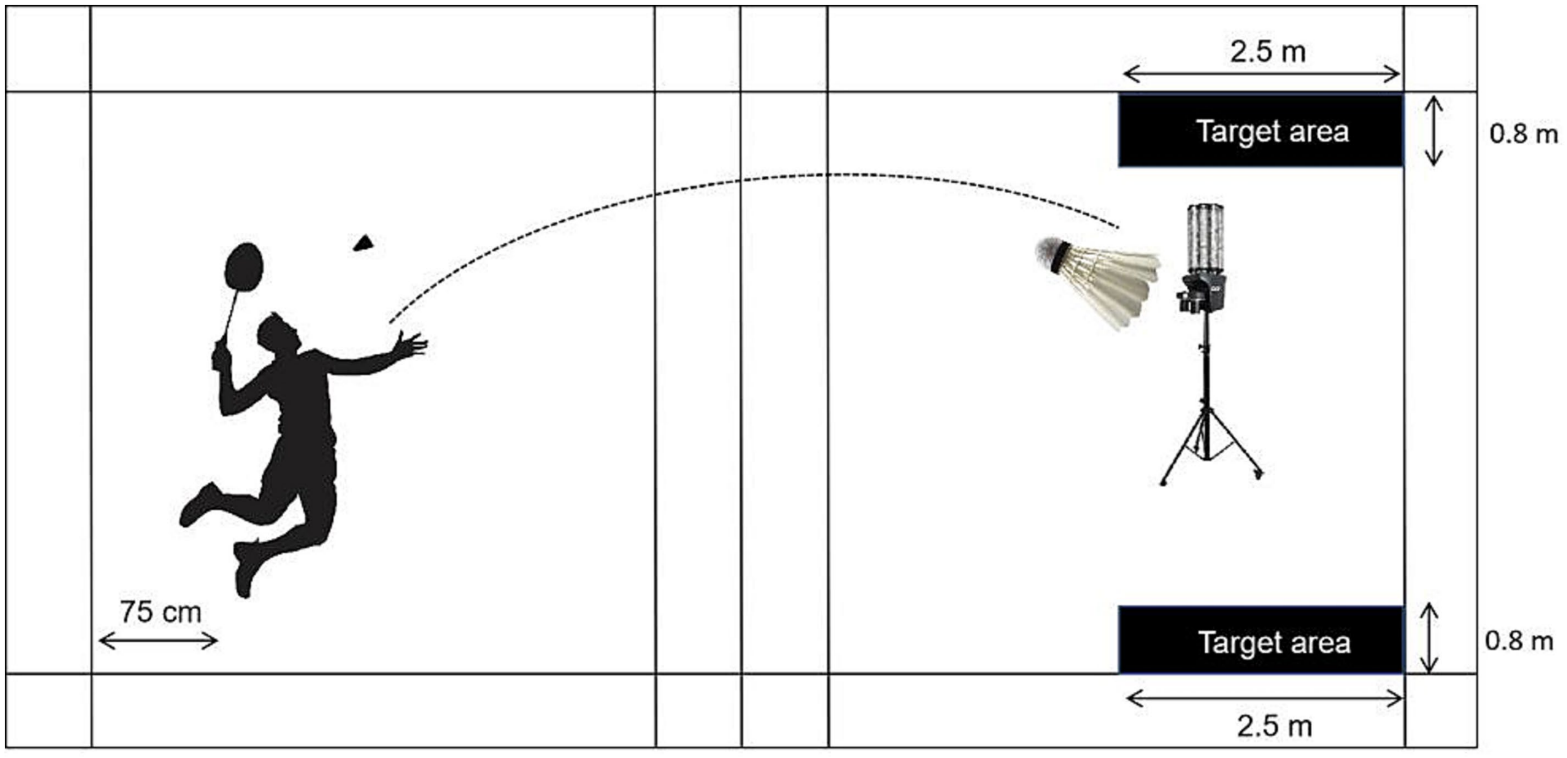
Schematic illustration of the Smash Accuracy Test (SAT).

Shuttlecocks were delivered by a shuttle feeder (Model 8,025, ASIBOSI, Dongguan, China) to ensure consistency in speed, trajectory, and placement, with each shuttle aimed at a designated position 75–80 cm inside the baseline (Figure 3). One shuttle was delivered every 3 s, for a total of 20 trials, and the number of successful smashes landing within the target zone was recorded. A new shuttle was used for each attempt, and any damaged shuttles were replaced immediately.

#### 2.3.3 Shuttle run agility test (SRAT)

**Figure 4:**
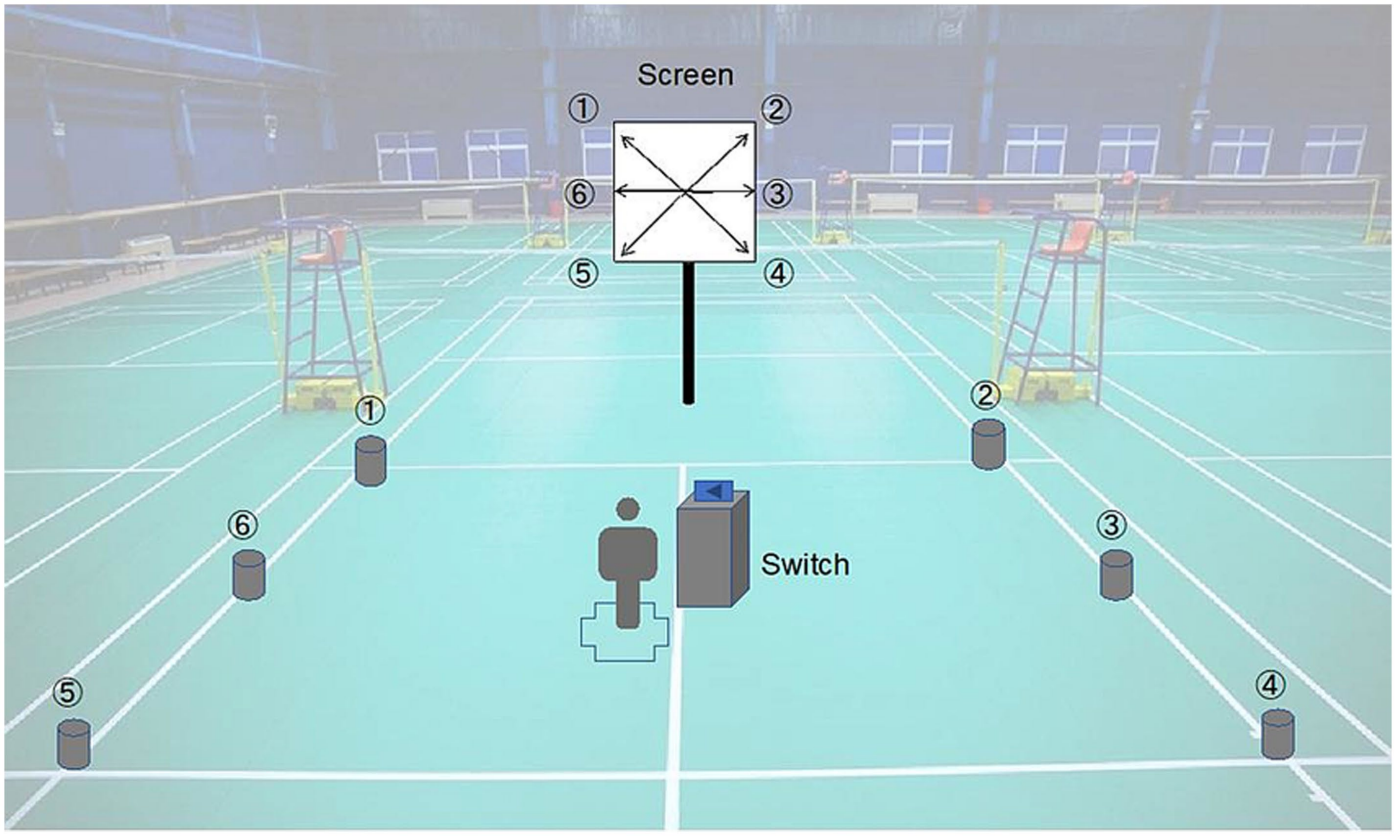
Schematic illustration of the Shuttle Run Agility Test (SRAT).

This test was adapted from the protocol described by Loureiro et al. (26) and conducted on a half badminton court. Six square target zones (0.2 m × 0.2 m) were marked on the floor (Figure 4). Participants stood at the central mark holding a control button, with a display screen positioned 2.5 m in front. The screen randomly presented an arrow pointing to one of the six target zones, with the sequence pre-set to avoid repetition.

Once the participant pressed the button, an arrow cue appeared, and the participant was required to sprint to the corresponding target zone, touch it, and immediately return to the starting point before initiating the next trial. Each set consisted of six repetitions, and three sets were performed with 20 s of passive rest between sets. The completion time of each set was recorded, and the fastest set was taken as the final performance outcome. Prior to testing, participants performed practice trials at a moderate pace until they were familiarized with the task.

#### 2.3.4 Repeated Sprint test (RST)

**Figure 5:**
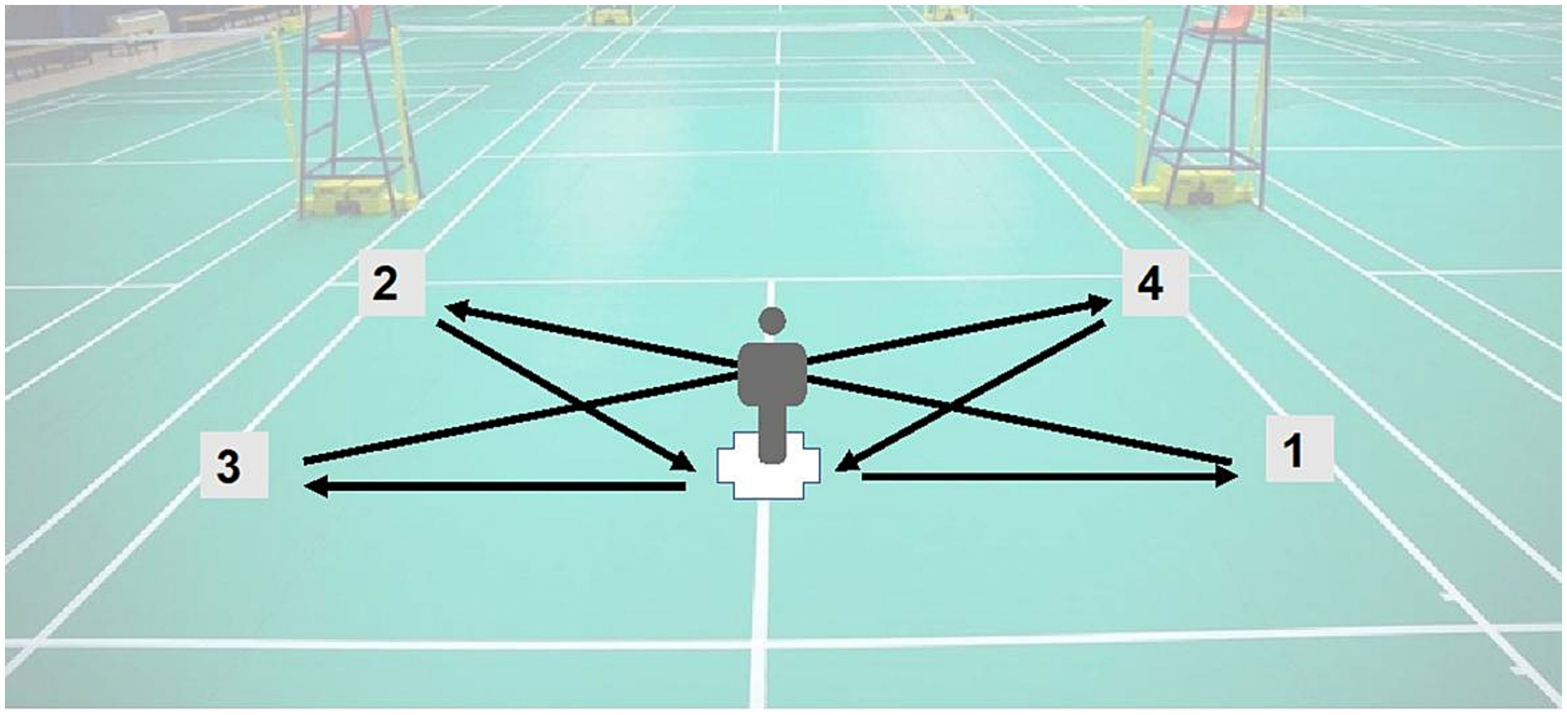
Schematic illustration of the Repeated Sprint Test (RST).

This test was designed to simulate the specific physical demands of badminton match play (27). The protocol consisted of two sets, each including 10 repetitions of 10-s sprints, with 20 s of passive recovery between sprints and a 3-min rest interval between sets. The sprints were performed on a predefined route on a badminton court (Figure 5). Participants were instructed to complete each sprint at maximal effort, and total distance and time for each set were recorded.

Participants were instructed to sprint along the designated route on the badminton court, following the sequence indicated by arrows from positions 1 to 4, and then return to the starting point.

#### 2.3.5 Fatigue simulation protocol

To replicate the physiological and technical demands of high-intensity badminton match play, a standardized 50-min training protocol was implemented. The session consisted of high-intensity footwork drills, sport-specific agility exercises, and intermittent 3-min walking recovery periods at 5.0 km/h, adapted from the methodology of Bottoms et al. (28). During the protocol, players wore a heart rate monitor (Polar V800, Polar Electro Oy, Finland) for continuous cardiac recording. Ratings of perceived exertion (RPE) were collected immediately before and after the protocol to assess subjective fatigue.

### 2.4 Statistical analysis

The primary outcome measures included anticipation accuracy, reaction time, AOI search frequency, shuttle run agility performance, repeated sprint distance, and smash accuracy. These variables were analyzed using linear mixed models (LMM). Fixed effects in the model included treatment (CAF vs. PLA), time (baseline vs. post-fatigue), sequence (CAF → PLA vs. PLA → CAF), and period order, with participants (Subject) treated as a random effect. To ensure the internal validity of the crossover design, sequence, period, and carryover effects were explicitly tested; none reached statistical significance (*p* > 0.1), suggesting that potential confounding effects could be disregarded.

In reporting the results, primary emphasis was placed on the treatment × time interaction effect, as it provides insight into whether caffeine differentially influenced performance under fatigue conditions. For variables with significant interactions, simple effects analyses were conducted, and 95% confidence intervals (CI) were reported.

For the secondary outcomes, including heart rate and ratings of perceived exertion (RPE), two-tailed paired *t*-tests were performed to compare differences between the two treatment conditions, as only single measurements were available. To control for the risk of Type I error in the context of multiple comparisons, Bonferroni correction was applied to adjust the significance threshold. All statistical tests were two-tailed, with significance set at *p* < 0.05.

All analyses were conducted using SPSS version 26.0 (IBM, USA), and figures were generated with GraphPad Prism version 8.0 (GraphPad Software, USA). Descriptive statistics are presented as mean ± standard deviation (SD), whereas LMM results are reported as estimated marginal means (EMM) with 95% confidence intervals (CI).

## 3 Results

### 3.1 Anticipation Skills Test (AST)

As one of the core outcomes of this study, a linear mixed model (LMM) was applied to evaluate the effects of caffeine on badminton players’ anticipation ability, including anticipation accuracy, reaction time, and visual search frequency in the areas of interest (AOI).

**Figure 6:**
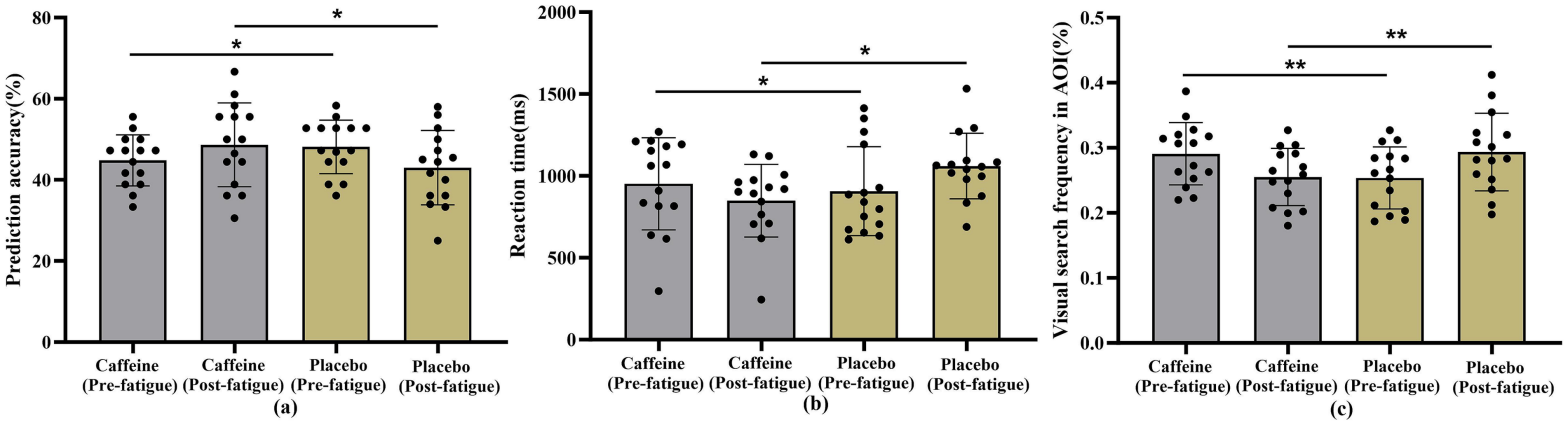
Results of the badminton-specific anticipation test. **(a)** Anticipation accuracy; **(b)** Reaction time; **(c)** AOI visual search frequency; *p < 0.05;** *p* < 0.01.

For anticipation accuracy (Figure 6a), neither the main effect of treatment (*F*(1,56) = 0.328, *p* = 0.569) nor time (*F*(1,56) = 0.087, *p* = 0.770) was significant. However, the treatment × time interaction was significant (*F*(1,56) = 4.503, *p* = 0.038). *Post hoc* analysis revealed that in the placebo condition, accuracy declined from a baseline of 48.1% (95% CI: 43.8–52.4%) to 42.9% (95% CI: 38.6–47.2%) following fatigue. In contrast, the caffeine condition showed an improvement from 44.8% (95% CI: 40.5–49.1%) at baseline to 48.7% (95% CI: 44.4–53.0%) post-fatigue, suggesting that caffeine ingestion counteracted fatigue-induced declines and exerted a positive effect.

Reaction time results (Figure 6b). Analysis revealed that the main effects of group (*F*(1,56) = 1.721, *p* = 0.195) and time (F(1,56) = 0.151, *p* = 0.699) were not significant. However, a significant group × time interaction was observed (*F*(1,56) = 4.084, *p* = 0.048). *Post hoc* analysis indicated that, in the placebo condition, reaction time increased from a baseline of 907.39 ms (95% CI: 780.24–1034.55) to 1060.33 ms (95% CI: 933.18–1187.48) following fatigue. In contrast, in the caffeine condition, reaction time decreased from 952.40 ms (95% CI: 825.25–1079.55) at baseline to 848.79 ms (95% CI: 721.64–975.94) after fatigue.

For AOI visual search frequency (Figure 6c), neither the main effect of treatment (F(1,56) = 0.002, *p* = 0.961) nor time (F(1,56) = 0.026, *p* = 0.872) was significant. However, a significant treatment × time interaction was observed (F(1,56) = 8.514, *p* = 0.005). Further analysis revealed that in the placebo condition, search frequency remained stable from baseline (0.3, 95% CI: 0.3–0.3%) to post-fatigue (0.3, 95% CI: 0.3–0.3%). In contrast, in the caffeine condition, search frequency decreased from 0.3% (95% CI: 0.3–0.3%) at baseline to 0.2% (95% CI: 0.2–0.3%) post-fatigue, suggesting that caffeine intake reduced unnecessary scanning and promoted more efficient visual search under fatigue.

### 3.2 Smash Accuracy Test (SAT)

**Figure 7:**
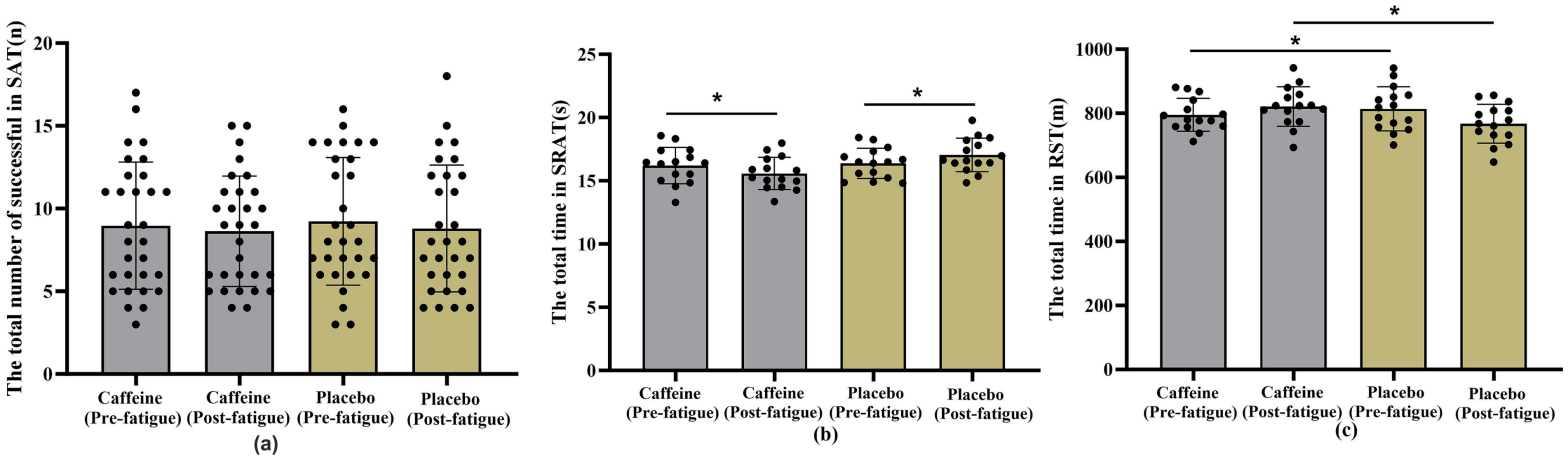
Results of smash accuracy and physical performance tests. **(a)** Smash Accuracy Test (SAT); **(b)** Shuttle Run Agility Test (SRAT); **(c)** Repeated Sprint Test (RST); *p < 0.05.

The results of the smash accuracy test are presented in Figure 7a. Linear mixed model analysis showed that the main effect of group was not significant (*F*(1,116) = 0.101, *p* = 0.751), indicating no overall difference in smash accuracy between the caffeine and placebo conditions. The main effect of time was also not significant (F(1,116) = 0.318, *p* = 0.574), suggesting no significant change in smash accuracy before and after fatigue. In addition, the group × time interaction was not significant (F(1,116) = 0.005, *p* = 0.942), indicating that caffeine ingestion did not significantly modulate the fatigue-induced changes in smash accuracy.

### 3.3 Shuttle Run Agility Test (SRAT)

The results of the shuttle run agility test are presented in Figure 7b. The linear mixed model revealed that the main effect of time was not significant (*F*(1,56) = 0.125, *p* = 0.725), indicating no overall difference in agility performance before and after fatigue. Similarly, the group × time interaction was not significant (*F*(1,56) = 2.352, *p* = 0.131). However, the main effect of group was significant (*F*(1,56) = 4.097, *p* = 0.048), showing that the caffeine group outperformed the placebo group overall. Further analysis indicated that, in the placebo group, completion time increased from a baseline of 16.38 s (95% CI: 15.67–17.09) to 17.05 s (95% CI: 16.34–17.76) after fatigue, reflecting a performance decline. In contrast, the caffeine group improved, with completion time decreasing from 16.02 s (95% CI: 15.49–16.91) at baseline to 15.78 s (95% CI: 15.07–16.50) after fatigue.

### 3.4 Repeated Sprint Test (RST)

The results of the repeated sprint test are presented in Figure 7c. The linear mixed model revealed that neither the main effect of group (F(1,56) = 1.195, *p* = 0.279) nor the main effect of time (F(1,56) = 0.444, *p* = 0.508) was significant. However, a significant group × time interaction was observed (F(1,56) = 5.283, *p* = 0.025). Further analysis showed that, in the placebo group, sprint distance decreased from 814.27 m (95% CI: 782.69–845.85) at baseline to 767.53 m (95% CI: 735.96–799.11) after fatigue, indicating a performance decline. In contrast, the caffeine group improved, with sprint distance increasing from 795.27 m (95% CI: 763.69–826.85) at baseline to 821.00 m (95% CI: 789.42–852.58) after fatigue.

### 3.5 Heart rate and ratings of perceived exertion (RPE)

**Figure 8:**
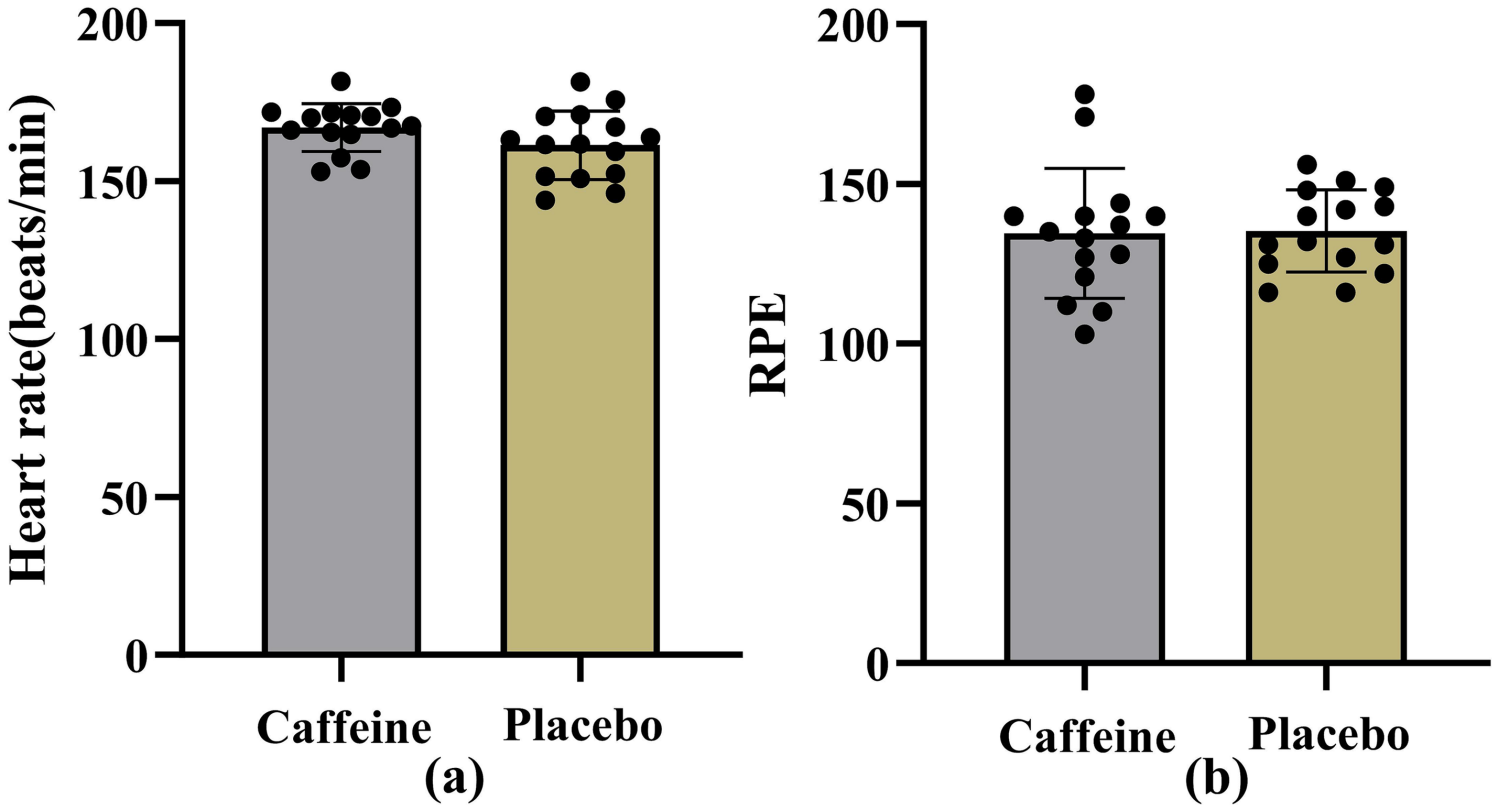
Heart rate and ratings of perceived exertion (RPE) results. 350 **(a)** Mean heart rate (beats/min) during the fatigue protocol; **(b)** Ratings of perceived exertion (RPE).

As shown in Figure 8a, independent t-test results indicated that the mean heart rate in the caffeine condition (166.50 ± 8.75 bpm) was slightly higher than in the placebo condition (161.86 ± 10.22 bpm), but the difference did not reach statistical significance (*p* = 0.079). Regarding ratings of perceived exertion (RPE), no significant difference was observed between treatments (*p* = 0.917). The mean RPE was 12.24 ± 1.78 in the caffeine group and 12.30 ± 1.12 in the placebo group (Figure 8b).

## 4 Discussion

This study employed a randomized, double-blind, crossover design and used linear mixed models to evaluate the acute effects of caffeine ingestion (5 mg/kg) on multiple dimensions of badminton-specific performance in elite players, including cognitive (anticipation accuracy, reaction time, visual search efficiency), physical (agility, repeated sprint ability), and technical (smash accuracy) domains. The findings indicate that caffeine significantly enhanced anticipation performance and sprint ability under fatigue, and was generally beneficial for overall agility performance, whereas no clear effects were observed for smash accuracy or ratings of perceived exertion (RPE). These results provide further evidence and boundary conditions for the application of caffeine in high-intensity, fast-decision racket sports.

Specifically, in the anticipation task, accuracy decreased after fatigue in the placebo group but increased in the caffeine group; likewise, reaction times lengthened with fatigue in the placebo condition but shortened under caffeine. This suggests that caffeine not only offset fatigue-induced declines in cognitive and behavioral performance but also enhanced decision speed and accuracy. These findings are consistent with prior evidence showing that caffeine, as a central nervous system stimulant, enhances alertness, attention, and executive control, thereby accelerating the perception–decision–action chain (29, 30, 50). In the fast-paced, high-intensity, and information-dense context of badminton, athletes must rapidly extract visual information, identify tactical cues, and execute motor programs; by improving attentional maintenance and information-processing speed, caffeine can help shorten reaction times and stabilize accuracy (31).

In line with this, the present study found that AOI search frequency decreased following fatigue under caffeine, while it remained largely unchanged with placebo, suggesting a shift from dispersed to more focused and efficient visual search. This likely reflects greater allocation of fixations to key cues (e.g., the opponent’s upper limbs, racket, or trunk), reducing ineffective scanning. These results converge with evidence that nutritional interventions can improve accuracy and reaction times under mental fatigue (32) and that caffeine can optimize visual search and perceptual–attentional processes (3). Overall, enhancements at the cognitive–attentional level appear to underlie caffeine’s ability to maintain or even improve anticipation and reaction performance under fatigue.

Regarding the physical performance outcomes, caffeine was found to significantly enhance overall agility levels, although no additional interaction effect was observed between pre- and post-fatigue conditions. This finding aligns with previous reports showing beneficial effects of caffeine on agility and change-of-direction performance (33), suggesting that improvements may stem from enhanced neuromuscular excitability and increased motor unit recruitment (33–35).

In the repeated sprint test (RST), sprint distance decreased after fatigue in the placebo group but increased in the caffeine group, reflecting a clear anti-fatigue pattern. This outcome is consistent with mechanisms whereby caffeine facilitates Ca^2+^ release within muscle fibers, enhances excitation–contraction coupling (36–38), augments sympathetic activation, and promotes fat oxidation and glycogen sparing (39, 40). For high-intensity sports characterized by frequent accelerations, decelerations, changes of direction, and short sprints (41, 42, 49), this anti-fatigue effect carries direct competitive relevance. Additionally, caffeine’s antagonism of adenosine receptors may reduce perceptions of effort and fatigue, thereby sustaining motor output during repeated high-intensity shuttle runs (43, 44).

It should be noted that although research specific to badminton remains limited, findings from other racket sports provide valuable external reference points for our results. For example, in tennis skill tests, ingestion of 6 mg/kg caffeine significantly improved shot success rates and accuracy (7, 10). These findings resonate with the present study’s outcomes on sprint performance and agility, suggesting that the ergogenic effects of caffeine may have cross-sport applicability across different racket disciplines.

In contrast to the cognitive and physical outcomes, no significant group, time, or interaction effects were observed for smash accuracy, suggesting that the acute influence of caffeine on fine motor execution is limited. This may be attributed to the highly automated and procedural nature of the smash action, whose stability depends more on long-term training and motor memory than on short-term stimulant effects (45, 46). Similar perspectives have been reported by Hadžimehmedović et al. (47), who noted that improvements in fine motor skills rely predominantly on sustained practice and technical adaptation rather than acute nutritional or pharmacological stimulation. Thus, the present findings further support the notion that caffeine is effective in enhancing cognition and physical performance but exerts limited immediate effects on highly skilled, automatized actions.

Moreover, with respect to heart rate and ratings of perceived exertion (RPE), this study found no significant differences between caffeine and placebo conditions, which may reflect considerable inter-individual variability in caffeine responsiveness. Such differences are influenced by genetic factors (e.g., CYP1A2 genotype), habitual caffeine consumption, and psychological expectations (48). For instance, Cornelis et al. demonstrated that genetic variability shapes both caffeine consumption and its physiological effects, implying that sensitivity to caffeine may modulate its impact on performance outcomes. Future research should therefore integrate genotyping and individual tolerance assessments to develop personalized supplementation strategies aimed at maximizing caffeine’s ergogenic potential. Tailored dosing protocols, adjusted for genetic background, could help clarify the extent to which caffeine optimally benefits sport-specific performance.

## 5 Limitations

Although this study systematically evaluated the multidimensional effects of acute caffeine ingestion on badminton-specific performance, several limitations should be acknowledged. (1) Only participants with low habitual caffeine intake (<50 mg/day) were included. Thus, the findings may not be generalizable to individuals with long-term or high habitual caffeine consumption, and future studies should verify the effects across different intake backgrounds. (2) The present work examined only acute effects; long-term supplementation or periodized ingestion was not addressed. Consequently, issues such as tolerance, cumulative effects, or rebound phenomena remain unclear. (3) The relatively small sample size, restricted to male elite badminton athletes, limits the external validity of the results, particularly regarding female athletes and players of different competitive levels. (4) The caffeine dose of 5 mg/kg adopted here is widely recommended in the literature and by the International Society of Sports Nutrition (ISSN) as an effective and safe dosage. However, no comparisons with other doses (e.g., 3 mg/kg or ≥6 mg/kg) were performed, preventing conclusions about dose–response effects in badminton-specific performance. (5) The study was not formally registered as a clinical trial, which may affect research transparency. (6) Although linear mixed models with Bonferroni corrections were employed, the use of multiple testing still carries a risk of Type I error, which should be addressed in future studies with larger samples and stricter analytical strategies.

## 6 Conclusion

This study demonstrates that acute ingestion of caffeine at a dose of 5 mg/kg body mass effectively enhances cognitive performance (anticipation accuracy, reaction time, and visual search efficiency) and physical performance (agility and sprint ability) in elite badminton players under fatigue conditions, while exerting limited influence on fine technical skills (smash accuracy) and perceived exertion. These findings suggest that caffeine may serve as a safe and effective ergogenic aid in the context of high-intensity, rapid-decision-making demands in badminton, with meaningful potential for practical application.

## Data Availability

The original contributions presented in the study are included in the article/supplementary material, further inquiries can be directed to the corresponding authors.

## References

[1] Da silva WF Lopes-Silva JP Camati Felippe LJ Ferreira GA Lima-Silva AE Silva-Cavalcante MD. Is caffeine mouth rinsing an effective strategy to improve physical and cognitive performance? A systematic review. Crit Rev Food Sci Nutr. (2023) 63:438–46. doi: 10.1080/10408398.2021.1949576, PMID: 34275371

[2] Grgic J Mikulic P. Effects of caffeine on rate of force development: a meta-analysis. Scand J Med Sci Sports. (2022) 32:644–53. doi: 10.1111/sms.14109, PMID: 34861076

[3] Wu S Chen Y Chen C Liu H Liu Z Chiu C. Caffeine supplementation improves the cognitive abilities and shooting performance of elite e-sports players: a crossover trial. Sci Rep. (2024) 14:2074. doi: 10.1038/s41598-024-52599-y, PMID: 38267565 PMC10808346

[4] Ricupero S Ritter FE. Caffeine and cognition: a cognitive architecture-based review. Theor Issues Ergon Sci. (2024) 25:655–79. doi: 10.1080/1463922X.2024.2323547

[5] Alshammary S Mohamed A. Caffeine intake among northern border area population in Saudi Arabia. Saudi J Med Pharm Sci. (2020) 6:77–90. doi: 10.36348/sjmps.2020.v06i01.013

[6] Kennedy D Wightman E. Mental performance and sport: caffeine and co-consumed bioactive ingredients. Sports Med. (2022) 52:69–90. doi: 10.1007/s40279-022-01796-8, PMID: 36447122 PMC9734217

[7] Cameron M Camic C Doberstein S Erickson J Jagim A. The acute effects of a multi-ingredient pre-workout supplement on resting energy expenditure and exercise performance in recreationally active females. J Int Soc Sports Nutr. (2018) 15:206. doi: 10.1186/s12970-017-0206-7 PMC5755346 29311763

[8] Kreutzer A Graybeal AJ Moss K Braun-Trocchio R Shah M. Caffeine supplementation strategies among endurance athletes. Front Sports Act Living. (2022) 4:821750. doi: 10.3389/fspor.2022.821750, PMID: 35463835 PMC9030507

[9] Salinero J Lara B Ruiz-Vicente D Areces F Puente-Torres C Gallo-Salazar C. Cyp1a2 genotype variations do not modify the benefits and drawbacks of caffeine during exercise: a pilot study. Nutrients. (2017) 9:269. doi: 10.3390/nu9030269 28287486 PMC5372932

[10] Domínguez R Veiga-Herreros P Sánchez-Oliver A Montoya J Ramos-Álvarez J Miguel-Tobal F. Acute effects of caffeine intake on psychological responses and high-intensity exercise performance. Int J Environ Res Public Health. (2021) 18:584. doi: 10.3390/ijerph18020584 33445587 PMC7827590

[11] Delleli S Ouergui I Messaoudi H Trabelsi K Ammar A Glenn J. Acute effects of caffeine supplementation on physical performance, physiological responses, perceived exertion, and technical-tactical skills in combat sports: a systematic review and meta-analysis. Nutrients. (2022) 14:2996. doi: 10.3390/nu14142996, PMID: 35889953 PMC9315598

[12] Szerej K Dorobek W Stankiewicz K Świeczkowski-Feiz J. The role of caffeine in enhancing physical performance: from metabolism to muscle function. J Educ Health Sport. (2024) 59:158–65. doi: 10.12775/jehs.2024.59.010

[13] Valldecabres R Trigueros ADB Sanjurjo CAC Abella CP. 2015 badminton world championship: singles final men's vs women's behaviours. J Hum Sport Exerc. (2017) 12:775–88. doi: 10.14198/jhse.2017.12.Proc3.01

[14] Laffaye G Phomsoupha M Dor F. Changes in the game characteristics of a badminton match: a longitudinal study through the Olympic game finals analysis in men's singles. J Sports Sci Med. (2015) 14:584–90. PMID: 26335338 PMC4541123

[15] Alder DB Broadbent DP Stead J Poolton J. The impact of physiological load on anticipation skills in badminton: from testing to training. J Sports Sci. (2019) 37:1816–23. doi: 10.1080/02640414.2019.1596051, PMID: 30931825

[16] Alder D Ford PR Causer J Williams AM. The coupling between gaze behavior and opponent kinematics during anticipation of badminton shots. Hum Mov Sci. (2014) 37:167–79. doi: 10.1016/j.humov.2014.07.002, PMID: 25222127

[17] Clarke N Duncan M. Effect of carbohydrate and caffeine ingestion on badminton performance. Int J Sports Physiol Perform. (2016) 11:108–15. doi: 10.1123/ijspp.2014-0426, PMID: 26024551

[18] Duncan M Stanley M Parkhouse N Cook K Smith M. Acute caffeine ingestion enhances strength performance and reduces perceived exertion and muscle pain perception during resistance exercise. Eur J Sport Sci. (2011) 13:392–9. doi: 10.1080/17461391.2011.635811, PMID: 23834545

[19] Grgic J Trexler ET Lazinica B Pedisic Z. Effects of caffeine intake on muscle strength and power: a systematic review and meta-analysis. J Int Soc Sports Nutr. (2018) 15:11. doi: 10.1186/s12970-018-0216-0, PMID: 29527137 PMC5839013

[20] Jodra P Lago-Rodríguez A Sánchez-Oliver AJ López-Samanes A Pérez-López A Veiga-Herreros P. Effects of caffeine supplementation on physical performance and mood dimensions in elite and trained-recreational athletes. J Int Soc Sports Nutr. (2020) 17:2. doi: 10.1186/s12970-019-0332-5, PMID: 31900166 PMC6942320

[21] Guest NS VanDusseldorp TA Nelson MT Grgic J Schoenfeld BJ Jenkins NDM. International society of sports nutrition position stand: caffeine and exercise performance. J Int Soc Sports Nutr. (2021) 18:1. doi: 10.1186/s12970-020-00383-4, PMID: 33388079 PMC7777221

[22] De Souza JG Del Coso J Fonseca FS Silva BVC de Souza DB da Silva Gianoni RL. Risk or benefit? Side effects of caffeine supplementation in sport: a systematic review. Eur J Nutr. (2022) 61:3823–34. doi: 10.1007/s00394-022-02874-3, PMID: 35380245

[23] Williams AM Davids K. Visual search strategy, selective attention, and expertise in soccer. Res Q Exerc Sport. (1998) 69:111–28. doi: 10.1080/02701367.1998.10607677, PMID: 9635326

[24] Wilson M Smith NC Chattington M Ford M Marple-Horvat DE. The role of effort in moderating the anxiety-performance relationship: testing the prediction of processing efficiency theory in simulated rally driving. J Sports Sci. (2006) 24:1223–33. doi: 10.1080/02640410500497667, PMID: 17176526

[25] Behan M Wilson M. State anxiety and visual attention: the role of the quiet eye period in aiming to a far target. J Sports Sci. (2008) 26:207–15. doi: 10.1080/02640410701446919, PMID: 17926174

[26] Loureiro L de Freitas PB. Development of an agility test for badminton players and assessment of its validity and test-retest reliability. Int J Sports Physiol Perform. (2016) 11:305–10. doi: 10.1123/ijspp.2015-0189 26217980

[27] Valenzuela PL Sánchez-Martínez G Torrontegi E Vázquez-Carrión J González M Montalvo Z. Acute responses to on-court repeated-sprint training performed with blood flow restriction versus systemic hypoxia in elite badminton athletes. Int J Sports Physiol Perform. (2019) 14:1280–7. doi: 10.1123/ijspp.2018-0878, PMID: 30958054

[28] Bottoms L Sinclair J Taylor K Polman R Fewtrell D. The effects of carbohydrate ingestion on the badminton serve after fatiguing exercise. J Sports Sci. (2012) 30:285–93. doi: 10.1080/02640414.2011.637948, PMID: 22176295

[29] Krishnan S Mohan S T J Manikandan S. Study of acute effect of caffeine on cognition among adults- an exploratory intervention trial. Med Legal Update. (2021) 21:50–5. doi: 10.37506/mlu.v21i4.3101

[30] Saville C Morree H Dundon N Marcora S Klein C. Effects of caffeine on reaction time are mediated by attentional rather than motor processes. Psychopharmacology. (2017) 235:749–59. doi: 10.1007/s00213-017-4790-7 29273820 PMC5847000

[31] Trunk A Stefanics G Zentai N Bacskay I Felinger A Thuróczy G. Effects of concurrent caffeine and mobile phone exposure on local target probability processing in the human brain. Sci Rep. (2015) 5:434. doi: 10.1038/srep14434, PMID: 26395526 PMC4585767

[32] Oliver L Sullivan J Russell S Peake J Nicholson M McNulty C. Effects of nutritional interventions on accuracy and reaction time with relevance to mental fatigue in sporting, military, and aerospace populations: a systematic review and meta-analysis. Int J Environ Res Public Health. (2021) 19:307. doi: 10.3390/ijerph19010307, PMID: 35010566 PMC8744602

[33] Stojanović E Scanlan A Milanović Z Fox J Stanković R Dalbo V. Acute caffeine supplementation improves jumping, sprinting, and change-of-direction performance in basketball players when ingested in the morning but not evening. Eur J Sport Sci. (2021) 22:360–70. doi: 10.1080/17461391.2021.1874059, PMID: 33413049

[34] Matsumura T Tomoo K Sugimoto T Tsukamoto H Shinohara Y Otsuka M. Acute effect of caffeine supplementation on 100-m sprint running performance: a field test. Med Sci Sports Exerc. (2022) 55:525–33. doi: 10.1249/mss.0000000000003057, PMID: 36251383 PMC9924959

[35] Ruíz-Moreno C Lara B Souza D Gutiérrez-Hellín J Romero-Moraleda B Cuéllar-Rayo Á. Acute caffeine intake increases muscle oxygen saturation during a maximal incremental exercise test. Br J Clin Pharmacol. (2020) 86:861–7. doi: 10.1111/bcp.14189 31782534 PMC7163369

[36] Mielgo-Ayuso J Marqués-Jiménez D Refoyo I Coso J León-Guereño P Calleja-González J. Effect of caffeine supplementation on sports performance based on differences between sexes: a systematic review. Nutrients. (2019) 11:2313. doi: 10.3390/nu11102313 31574901 PMC6835847

[37] Neyroud D Cheng A Donnelly C Bourdillon N Gassner A Geiser L. Toxic doses of caffeine are needed to increase skeletal muscle contractility. Am J Phys Cell Phys. (2019) 316:C246–51. doi: 10.1152/ajpcell.00269.2018, PMID: 30566390

[38] Pérez-López A Garriga-Alonso L Montalvo-Alonso J Val-Manzano M Valadés D Vila H. Sex differences in the acute effect of caffeine on repeated sprint performance: a randomized controlled trial. Eur J Sport Sci. (2024) 25:233. doi: 10.1002/ejsc.12233 PMC11680550 39662990

[39] Grgić J. Caffeine ingestion enhances Wingate performance: a meta-analysis. Eur J Sport Sci. (2017) 18:219–25. doi: 10.1080/17461391.2017.1394371 29087785

[40] Wang Z Qiu B Gao J Coso J. Effects of caffeine intake on endurance running performance and time to exhaustion: a systematic review and meta-analysis. Nutrients. (2022) 15:148. doi: 10.3390/nu15010148 36615805 PMC9824573

[41] Karayiğit R Forbes S Osmanov Z Demirtaş C Yaşli B Naderi A. Low and moderate doses of caffeinated coffee improve repeated sprint performance in female team sport athletes. Biology. (2022) 11:1498. doi: 10.3390/biology11101498, PMID: 36290401 PMC9598515

[42] Stojanović E Stojiljković N Scanlan A Dalbo V Stanković R Antić V. Acute caffeine supplementation promotes small to moderate improvements in performance tests indicative of in-game success in professional female basketball players. Appl Physiol Nutr Metab. (2019) 44:849–56. doi: 10.1139/apnm-2018-0671, PMID: 30633542

[43] Clarke N Baxter H Fajemilua E Jones V Oxford S Richardson D. Coffee and caffeine ingestion have little effect on repeated sprint cycling in relatively untrained males. Sports. (2016) 4:45. doi: 10.3390/sports4030045, PMID: 29910293 PMC5968880

[44] López-Torres O Rodríguez-Longobardo C Capel-Escoriza R Fernández-Elías V. Ergogenic aids to improve physical performance in female athletes: a systematic review with meta-analysis. Nutrients. (2022) 15:81. doi: 10.3390/nu15010081, PMID: 36615738 PMC9823656

[45] Grgić J Mikulić P. Caffeine ingestion acutely enhances muscular strength and power but not muscular endurance in resistance-trained men. Eur J Sport Sci. (2017) 17:1029–36. doi: 10.1080/17461391.2017.1330362, PMID: 28537195

[46] Pickering C Kiely J. Are the current guidelines on caffeine use in sport optimal for everyone? Inter-individual variation in caffeine ergogenicity, and a move towards personalised sports nutrition. Sports Med. (2017) 48:7–16. doi: 10.1007/s40279-017-0776-1, PMID: 28853006 PMC5752738

[47] Hadžimehmedović D Likić S Biščević I. Fine motor skills and BMI are good predictors of athletic performance in college students. Multidisciplinarni Pristupi Edukaciji I Rehabilitaciji. (2023) 5:140–6. doi: 10.59519/mper5208

[48] Cornelis M Monda K Yu K Paynter N Azzato E Bennett S. Genome-wide meta-analysis identifies regions on 7p21 (ahr) and 15q24 (cyp1a2) as determinants of habitual caffeine consumption. PLoS Genet. (2011) 7:e1002033. doi: 10.1371/journal.pgen.1002033, PMID: 21490707 PMC3071630

[49] Girard O Millet GP. Neuromuscular fatigue in racquet sports. Phys Med Rehabil Clin N Am. (2009) 20:161–73. doi: 10.1016/j.pmr.2008.10.008, PMID: 19084769

[50] Grgic J Grgic I Pickering C Schoenfeld BJ Bishop DJ Pedisic Z. Wake up and smell the coffee: caffeine supplementation and exercise performance-an umbrella review of 21 published meta-analyses. Br J Sports Med. (2020) 54:681–8. doi: 10.1136/bjsports-2018-100278, PMID: 30926628

